# Nutritional strategies against dementia in rural populations

**DOI:** 10.3389/fnut.2025.1677197

**Published:** 2025-12-15

**Authors:** Bruno S. Goncalves, Naga L. Gannavaram, Cohen Yates, Aishniya Kandula, Aleen Nayyar, Sneha S. Pillai, Ridhi K. Puri, Asma Nayyar

**Affiliations:** 1 Departments of Surgery, Internal Medicine and Biomedical Sciences, Joan C. Edwards School of Medicine, Marshall University, Huntington, WV, United States; 2 Department of Medicine, Sharif Medical and Dental College, Lahore, Pakistan

**Keywords:** dementia, cognitive decline, gut-brain axis, rural population, nutraceuticals

## Abstract

Dementia is a progressive neurodegenerative disorder that represents a growing challenge to global health, especially in aging populations. The burden of dementia is high in rural communities, where access to healthcare services, nutritional resources, and educational opportunities is significantly limited. A critical knowledge gap exists regarding how rural-specific nutritional disparities mechanistically interact with gut–brain axis dysfunction to influence dementia risk. The gut-brain axis mediates neuroimmune communication, metabolic regulation, and microbial signaling, and nutritional insufficiency is associated with reduced microbial diversity, impaired short-chain fatty acid (SCFAs) production, increased intestinal permeability, and heightened systemic inflammatory activity, all of which contribute to neurodegenerative processes. This review delineates the mechanistic pathways linking malnutrition-induced gut dysregulation to neuronal injury and cognitive decline, with a specific focus on rural populations. We further evaluate the biological actions of key nutraceutical classes, including phytochemicals (curcumin, resveratrol, sulforaphane, ginkgo biloba), vitamins (B12, C, E, and D), and metabolic/dietary interventions (omega-3 fatty acids, ketogenic diet, Mediterranean diet, trehalose). By consolidating preclinical and clinical evidence, we identify the molecular targets through which these nutraceuticals modulate oxidative stress, inflammatory signaling networks, blood-brain barrier (BBB) integrity, and microRNA regulation. In summary, our findings suggest that nutraceutical-based approaches targeting gut-brain axis dysfunction may provide a feasible and biologically informed strategy for mitigating dementia disparities in rural settings.

## 1 Introduction

Dementia is a major health problem worldwide, involving progressive cognitive decline that interferes with independent daily living (1). Common subtypes include vascular dementia, frontotemporal dementia, Lewy body dementia, mixed dementia, and Alzheimer's disease (AD) (1, 2). The lifetime risk for dementia in individuals over 55 years old in the U.S. is 42%, with elevated risk in females, black adults, and APOE4 gene carriers (3). Studies have shown that rural residents are disproportionately underdiagnosed, diagnosed at advanced stages, and experience a disproportionate mortality burden from dementia-related causes (4–6). Even without a diagnosis of dementia, older adults in rural populations exhibit reduced cognitive performance along a rural-urban gradient, a disparity that is mechanistically linked to structural socioeconomic determinants such as attenuated educational attainment, a higher burden of chronic health conditions, and persistent socioeconomic stressors, including food and resource insecurity (7–10).

Epidemiological studies show that rural populations experience a disproportionate prevalence of cognitive impairment and dementia compared with urban areas. In the United States, older adults living in rural settings exhibit accelerated cognitive decline across the rural-urban gradient, in part due to attenuated educational attainment, higher rates of chronic illness, reduced healthcare access, and limited availability of nutritious foods (4, 5). Rural communities also demonstrate elevated levels of food insecurity and poorer dietary quality, both of which contribute to systemic inflammation and metabolic dysfunction (11).

In addition to traditional risk factors (hypertension and obesity), rural living is associated with increased nutritional inequities (8–10, 12, 13). Furthermore, nutritional disparity in the rural population is further exacerbated by geographic and economic barriers to accessing healthy foods (13, 14). The limited access to grocery stores, high food prices, and the dominance of ultra-processed foods (UPF) collectively reinforce a structural nutritional disadvantage (13). Roughly 2.3 million rural Americans live in “food deserts,” characterized by limited access to fresh fruit and vegetables (11, 15, 16). These environmental and economic constraints contribute to an inequitable risk profile for dementia in these communities.

Given the importance of identifying effective interventions for populations experiencing disproportionate dementia burden, the incorporation of nutraceuticals offers a promising pathway. The central objective of this review article is to provide a comprehensive overview of the current understanding of dementia in rural populations, its underlying pathophysiological mechanisms, and the potential therapeutic benefits of nutraceuticals. Briefly, the first section outlines how rural structural conditions create contextual disparities that increase vulnerability to cognitive decline. The following section examines the gut–brain axis to illustrate how nutritional patterns influence microbial metabolites and immune signaling. Subsequent sections discuss emerging evidence on nutraceuticals, with a detailed description of four potent phytochemicals, vitamins, and dietary/metabolic interventions with documented neuroprotective properties, including findings from human clinical trials.

This review extends beyond previous studies by presenting a unified view of how rural environmental and socioeconomic conditions intersect with dietary patterns and cognitive vulnerability. Unlike prior studies that consider diet or microbiota separately, these determinants are evaluated within the structural context of rural health, marked by food insecurity, limited healthcare access, and environmental exposures, to clarify their contribution to disparities in cognitive outcomes. This perspective underscores the need for approaches that consider both the social context and the biological pathways involved in cognitive decline.

It is important to clarify how rural environmental and nutritional disadvantages translate into measurable biological disruption. Nutritional inadequacies characteristic of rural settings, such as low fiber intake, micronutrient deficiencies, and reliance on ultra-processed foods (UPF), alter gut microbial composition, impair intestinal barrier integrity, and amplify systemic inflammatory signaling. These diet-induced perturbations constitute the mechanistic link between structural socioeconomic disadvantages and neurodegenerative processes, establishing the rationale for examining rural health disparities and their molecular consequences.

## 2 Rural health disparities and dementia risk

Nutrient deficiencies in rural populations significantly contribute to dementia risk through interconnected biological and social mechanisms. Chronic malnutrition, geographic isolation, and limited access to fresh, nutritious food create a cycle that exacerbates cognitive vulnerability (17). According to the United States Department of Agriculture, rural food deserts (areas with inadequate access to grocery stores) affect over 2.3 million Americans, disproportionately impacting older adults and low-income families (15). Food cost and geographic proximity are the main factors influencing food choices in rural Appalachia (13, 15). Individuals in these areas frequently consume energy-dense but nutrient-poor diets, leading to higher caloric intake and increased risk of chronic disease and neurodegeneration (13). A cross-sectional study revealed increasing reliance on packaged food products, further depleting diet quality (18). These intersecting structural determinants, including rurality, income level, food access, and self-care capacity, collectively intensify nutritional vulnerability and contribute to a disproportionate dementia burden.

Food illiteracy and comorbid health conditions further compound these disparities. Between 2001 and 2018, the average intake of minimally processed foods declined significantly (19). Rural adults consuming low-nutrient-dense diets were found to be twice as likely to be obese and deficient in vitamin B12, and up to 17 times more likely to fall short in essential nutrient intake (20). A randomized controlled trial demonstrated that a 2-week high ultra-processed food (UPF) diet caused substantial increases in energy intake and weight gain (21). Malnutrition represents an additional factor contributing to cognitive vulnerability in rural populations. Insufficient intake of nutrients, including omega-3 fatty acids, B-vitamins, and antioxidant compounds, has been linked to impaired synaptic function, increased oxidative stress, and increased neuroinflammation (22–24). Observational studies also show that older adults with nutrient deficiencies or poor diet quality have an elevated risk of cognitive decline and dementia (17, 25).

In the United States, significant rural-urban gradients in dementia burden have been shown, caused by differences in socioeconomic status, educational attainment, and dietary access. Older adults in rural areas show disproportionate prevalence of cognitive impairment and dementia compared with their urban counterparts, partly due to lower educational opportunities, higher poverty rates, and limited access to healthy foods and healthcare services (5, 26, 27).

Evidence suggests a strong correlation between diet quality and the risk of dementia (28). A prospective cohort study linked greater UPF intake with increased dementia incidence, while replacing UPFs with whole or minimally processed foods was associated with a 19% reduction in dementia risk (28). Chronic low-grade systemic inflammation, frequently amplified by the pro-inflammatory Standard American Diet prevalent in rural settings, is a central driver of neurodegenerative processes (29). This inflammatory state activates adipose and immune signaling pathways, which are critical components of the gut-brain axis. These effects are further exacerbated in rural communities, where heightened pollution exposure and lower antioxidant intake reflect environmental and nutritional inequities (30). Healthy Eating Index-2020 (HEI-2020) and the Dietary Inflammatory Index (DII) analyses revealed significant associations between elevated interleukin (IL)-6, IL-1β, lipopolysaccharide binding protein, Toll-like receptor 4 (TLR4), as well as zonula occludens-1 (ZO-1) and cognitive impairment, highlighting inflammation as a key modifiable target (31). Together, these biomarkers indicate a diet-linked inflammatory cascade in which impaired gut-barrier integrity facilitates microbial translocation, upregulates TLR4-mediated signaling, and intensifies peripheral immune activation. This inflammatory activity communicates with the brain, promoting microglial activation and reinforcing the neuroinflammatory processes associated with cognitive decline (31).

The absence of fruits, vegetables, whole grains, and lean proteins limits neuroprotective nutrients crucial for synaptic plasticity and energy metabolism (32). Polyphenols, omega-3 fatty acids, B-complex vitamins, and minerals support neurogenesis, membrane fluidity, and neurotransmission. However, high intake of palmitic acid and saturated fats disrupts the blood-brain barrier (BBB) and promotes β-amyloid accumulation (33). Deficiencies in vitamin B12 and omega-3s, especially docosahexaenoic acid (DHA), an essential component of synaptic membranes, contribute to oxidative stress, impaired signaling, and cognitive decline (34, 35).

Emerging evidence shows a significant global rise in early-onset dementia, emphasizing the impact of nutritional and metabolic factors throughout life. Global Burden of Disease data indicate that the number of individuals under 70 with dementia has been increasing, with continued growth projected through 2050 (36, 37). These findings highlight that nutritional interventions are relevant not only for older adults but also for preventing or slowing premature cognitive decline in younger populations, particularly in rural areas undergoing rapid dietary changes.

It has been shown that the gut-brain axis has a role in cognitive health (38, 39). Disruptions in this bidirectional system, including elevated inflammatory markers and compromised gut barrier integrity, are increasingly recognized as contributors to neurodegenerative processes. Elevated inflammatory markers and compromised gut integrity, reflected by increased intestinal permeability and dysbiosis, have been linked to cognitive impairment (31). The gut-brain connection mediates neuroinflammation through microbial metabolites and immune signaling, indicating that altered gut physiology represents a mechanistic pathway through which rural nutritional inequities contribute to cognitive decline (40). In this context, understanding how gut-brain signaling influences inflammatory and metabolic pathways clarifies why these mechanisms represent key targets for intervention in cognitive decline.

## 3 The gut-brain axis in cognitive decline

The gut microbiota plays a crucial role in maintaining systemic and neurological health (40). The gut-brain axis is a bidirectional communication network connecting the gut microbiome with the central nervous system through neural, immune, and endocrine pathways (41–43). Notably, the vagus nerve detects microbial metabolites, such as short-chain fatty acids (SCFAs), and relays these signals to the brain (44). These microbial metabolites, including amino acids and neurotransmitter precursors, influence both gastrointestinal and brain function. Disruption of the gut microbiota, known as gut dysbiosis, can impair neurotransmitter synthesis, compromise the intestinal barrier, and promote neuroinflammation, contributing to neurodegeneration, leading to cognitive impairment (45). Dysbiosis alters microglial activation, elevates pro-inflammatory cytokines including IL-6, IL-1β, and tumor necrosis factor-alpha (TNF-α), and reduces the production of beneficial microbial metabolites such as SCFAs (46, 47). SCFAs such as acetate, butyrate, and propionate produced by bacterial fermentation of dietary fiber have potent anti-inflammatory effects. Additionally, SCFAs regulate microglial function, suppress Nuclear Factor kappa-light-chain-enhancer of activated B cells (NF-κB) activation, and modulate both intestinal and blood-brain barrier integrity (48–50). Experimental models show that SCFA supplementation in Alzheimer's mice improves memory and reduces Aβ plaque formation by upregulating IL-10 and downregulating IL-6 (51, 52).

Several studies demonstrate that dietary patterns modulate gut-brain signaling (53–56). Diets rich in fiber, polyphenols, probiotics, and unsaturated fats increase beneficial SCFA-producing genera such as *Faecalibacterium* and *Roseburia*, resulting in elevated butyrate and propionate levels that suppress NF-κB activation, regulate microglial homeostasis, and reduce neuroinflammation (57, 58). However, high-fat or ultra-processed diets impair the microbiome, reducing SCFA production, increasing systemic pro-inflammatory cytokine (IL-6 and TNF-α), leading to gut permeability (through the downregulation of Occludin and E-cadherin), and promoting hippocampal neuroinflammation, which accelerates cognitive decline (59, 60). Food insecurity and nutritionally inadequate diets, which disproportionately affect rural communities, further compound these disruptions (61). These diet-induced alterations in gut microbial composition and metabolic activity can be quantitatively assessed through changes in microbial α- and β-diversity, circulating levels of SCFAs, and systemic inflammatory mediators. Together, these indicators provide a mechanistic link between nutritional patterns, microbiota-derived signaling, neuroinflammatory cascades, and the progression of neurodegenerative pathology (60, 62).

Emerging therapeutic approaches, such as probiotic supplementation, prebiotic fiber, and fecal microbiota transplantation (FMT), show promise in delaying cognitive decline (63, 64). In clinical trials, AD patients receiving multispecies probiotics exhibited improved cognition (65, 66). In addition, FMT in transgenic AD mouse models reduced inflammation via the TLR4-Myeloid differentiation factor 88 (MyD88)-NF-κB (TLR4/MyD88/NF-κB) pathway and increased SCFA production (67).

**Figure 1:**
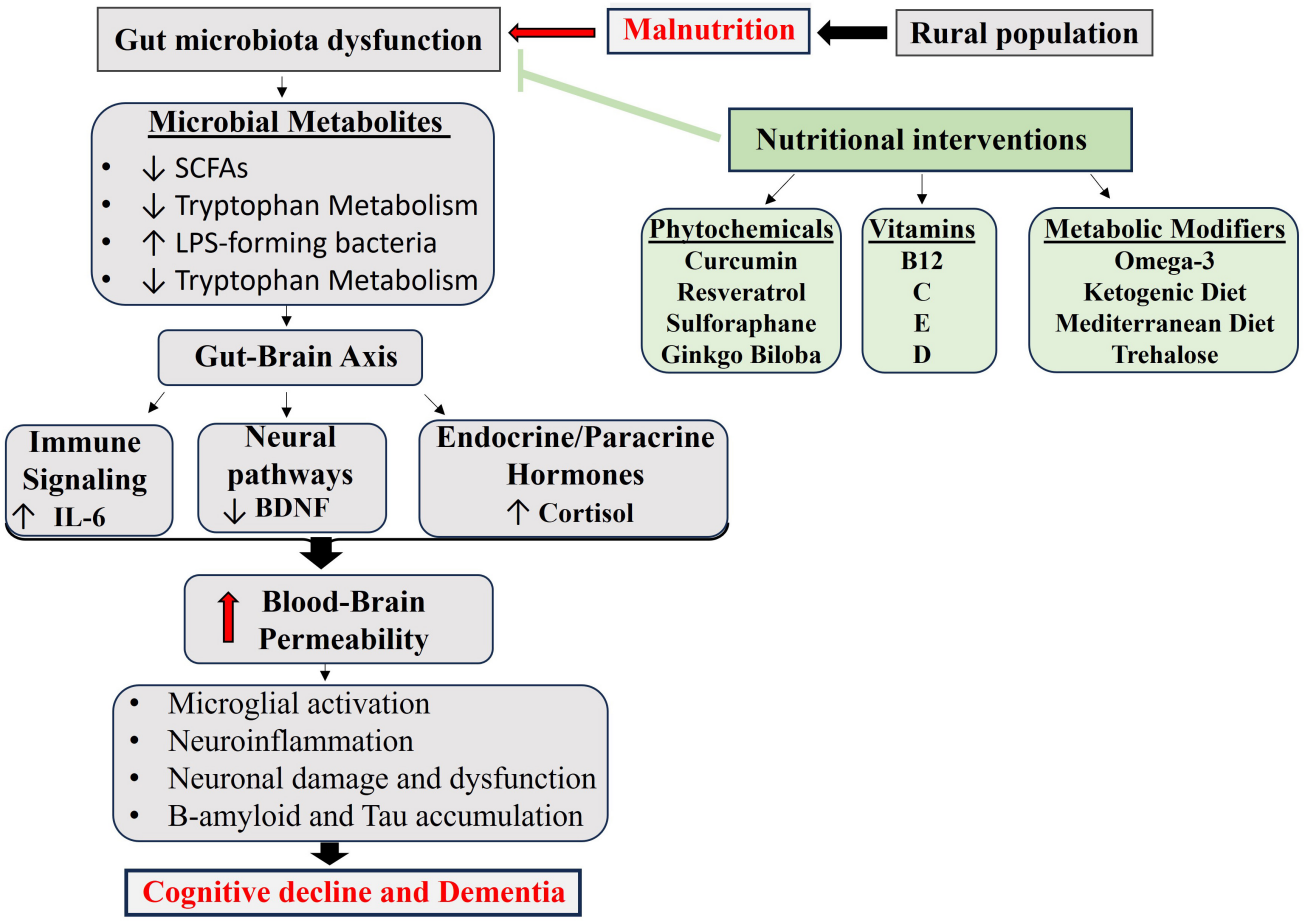
Schematic representation showing the protective effects of nutraceuticals in the dementia-associated gut-brain axis. Nutraceuticals effectively inhibit neuroinflammation and neurodegeneration through their remarkable pharmacological properties. Image proposed by the authors.

Environmental and social determinants, including pesticide exposure and access to healthcare, significantly impact gut microbiome health. Rural populations, particularly those in agricultural and resource-limited regions, experience disproportionate exposure to environmental toxins and have restricted access to healthcare and nutritious foods, making them especially susceptible to microbiome disruption (68, 69). These factors exacerbate systemic inflammation, reduce the availability of neuroprotective metabolites, and contribute to disease progression. Studies show that rural residents with dementia have shorter survival rates and a disproportionately higher disease burden compared with their urban counterparts (26, 70). However, there is no evidence directly comparing whether malnutrition or pesticide exposure has a greater impact on dementia risk. It has been demonstrated that both factors negatively impact gut-brain axis regulation by increasing oxidative stress, inflammation, and microbiome disruption. In rural areas, where these exposures frequently coexist, their combined effects may further contribute to contextual cognitive vulnerability (68, 69). Addressing microbiome health through targeted dietary strategies and nutraceutical interventions could offer a viable path to support cognitive resilience in these at-risk populations (Figure 1).

Taken together, this section shows a mechanistic pathway that has not been fully explored in previous studies. Rural environmental and socioeconomic determinants influence dietary quality and exposure patterns, altering gut microbial composition, metabolic signaling, and neuroinflammatory activation. Rural living conditions, therefore, represent a biologically relevant context in which disruptions to gut-brain communication arise, linking dietary insufficiency, microbiota imbalance, and immune activation to increased susceptibility to neurodegenerative processes. These mechanistic connections underscore the need for interventions capable of modulating inflammatory, metabolic, and microbiota-derived pathways in populations experiencing disproportionate dementia burden.

## 4 Nutraceuticals: mechanisms, and neuroprotective potential

Nutraceuticals, derived from foods and medicinal plants, have emerged as promising adjunctive strategies for the prevention and management of dementia (71, 72). Their therapeutic potential stems from the ability to modulate key molecular pathways implicated in neurodegeneration, including the suppression of pro-inflammatory cascades (73, 74). In addition, nutraceuticals influence synaptic plasticity and neuronal survival, which is crucial for memory and learning. Additionally, evidence highlights their role in modulating the expression of specific microRNAs (miRs), small non-coding RNAs that regulate gene networks involved in neuroinflammation (75–77). This multimodal activity positions nutraceuticals as attractive, low-cost interventions capable of improving cognitive decline, especially in vulnerable rural populations where conventional therapeutic options may be limited.

### 4.1 Phytochemicals

Phytochemicals are plant-derived compounds that can target neuroinflammation by reducing systemic inflammation, preserving the BBB, and protecting against gastrointestinal signaling to the brain (70, 78). It can interact with various signaling pathways to inhibit inflammation caused by Aβ peptide accumulation. By inhibiting pro-inflammatory gene expression, polyphenols can decrease neurodegeneration (79, 80). This section aims to analyze how the following phytochemicals- Curcumin, Resveratrol, Sulforaphane, and Ginkgo Biloba can modulate different signaling pathways and miRs to improve memory and cognitive functions. Table 1 summarizes these mechanisms.

**Table 1:** Effect of phytochemicals on the pathophysiological complications associated with cognitive decline/dementia.

**Phytochemical**	**Sources**	**Experimental models**	**Physiological effects**	**References**
Curcumin	Turmeric; Curcuma Longa Linn plant	*In *vitro**: Mononuclear cells. Astroglial cells SH-SY5Y cells BV2 cells Microglial cells *In *vivo**: Tg2576 mice APP/PS1 mice Clinical trials: Randomized, double-blind study Longitudinal study *In *Silico*:* Computational methods	↓ Aβ aggregation ↓ptau ↓ oxidative stress ↓ inflammation ↓HMGB1-RAGE/TLR4-NF-κB ↓BACE-1 ↓ ERβ and NFκB ↓ pERK, JNK, p38 ↓ pSTAT1/3 and JAK1/2 ↓ proinflammatory miR levels: miR146a, hsa-miR-26a-5p, hsa-miR-203a-3p, hsa-miR-155-5p ↑ BDNF levels ↑ phagocytosis	(86–94, 96, 97, 99–103, 305, 306)
Resveratrol	Cruciferous vegetables: broccoli, cauliflower, and brussels sprouts	*In *Vitro**: H19-7 cells. Astrocyte cells. RAW 264.7 cells Neural cells PC12 cells SH-SY5Y cells, HEK293 cells *In Vivo*: C57BL/6 mice CA1 mouse model Type II Diabetes Rats Clinical Trial: Double-blind, placebo-controlled study	↓ oxidative stress ↓ inflammation ↓ NOS, HO-1 ↓ lipid peroxidation ↓ apoptosis ↓ Aβ aggregation ↓ TLR4/NF-κB ↓ activity of STAT1/STAT3 ↓ ROCK1 ↓ metalloproteinase 9 ↑ SIRT1 ↓ miR-9-3p, miR-134, miR124 ↑ miR-146a ↑ pAMPK ↑ CREB synthesis ↑ BDNF activity	(81, 109, 110, 112–121, 123–125, 307, 308)
Sulforaphane	Cruciferous vegetables: broccoli, cauliflower, and brussels sprouts	*In vitro*: Microglial cells. SH-SY5Y cells. Microvascular endothelial cells *In vivo*: Vascular cognitive impairment induced rat models. PS1V97L mice *In *silico:** Computational docking modeling	↓ oxidative stress ↓ inflammation ↓ JNK/AP-1/NF-κB activity ↓ Aβ aggregation ↓ BACE-1 ↓Pro-inflammatory miR: miR-155, hsa-miR-17-5p, hsa-miR-16-5p, and hsa-miR-26b-5p ↑anti-inflammatory miR: miR-223 ↓ apoptosis ↑ Nrf2 signaling ↑ glutathione synthesis ↑ glucose transporter-1	(126, 131–143)
Ginkgo Biloba	Leaves, seeds, exocarp of the Chinese maidenhair tree/Ginkgo biloba tree	*In *vivo**: Tg2576 mouse; APP/PS1 mouse. Rats Clinical Trials: Double-blind, randomized placebo study	↓ Aβ aggregation ↓ptau ↓ oxidative stress ↓ inflammation ↓ APP levels ↓Akt/JNK/caspase-3 activity ↓proinflammatory miR: miR-1-3p/miR-206-3p ↑ dopamine signaling ↑antioxidant enzymes ↑ modulation of the cholinergic system	(145, 149, 153–158, 161)

Aβ aggregation, Beta-amyloid, HMGB1, High Mobility Group Box 1; RAGE, Receptor for Advanced Glycation End-products; TLR4, Toll-Like Receptor 4; NF-κB, Nuclear Factor kappa-light-chain-enhancer of activated B cells; BACE-1, Beta-site APP Cleaving Enzyme 1; ERβ, Estrogen Receptor β; p, phosphorylation; ERK, Extracellular signal-Regulated Kinase; JNK, c-Jun N-terminal Kinase; JAK, Janus Kinases; STATs, Signal Transducers and Activators of Transcription; BDNF, Brain-Derived Neurotrophic Factor; Nrf2, nuclear factor erythroid 2-related factor 2; HO-1, Heme oxygenase; NOS, Nitric oxide synthase; NLRP3, Nucleotide-binding domain, Leucine-rich Repeat, and Pyrin domain-containing protein 3; ROCK1, Rho-associated coiled-coil containing protein kinase 1; RANTES, Regulated on Activation, Normal T Cell Expressed and Secreted; CREB, cAMP Response Element-Binding Protein.

#### 4.1.1 Curcumin

Curcumin is a polyphenol compound extracted from the herb Curcuma Longa Linn (turmeric) (81). Curcumin is the active component in turmeric, which is a spice used in many Middle Eastern and South Asian diets in the Indian subcontinent (82). It is often consumed as an oral capsule, powder form, and it can be effective against a wide variety of chronic inflammatory conditions, such as dementia (83). Despite its therapeutic benefits, a major limitation of curcumin supplementation is its low systemic bioavailability due to rapid metabolism (84). In the context of neurodegeneration, curcumin protects against dementia by scavenging reactive oxygen species (ROS), suppressing pro-inflammatory cytokines, and modulating signaling pathways (85). It improves cognitive function, spatial memory, and decreases AD-associated pathology, such as tubulin associated unit (tau) phosphorylation and Aβ plaques (86).

Through multiple studies, curcumin has exhibited an array of activity against dementia progression (87–90). An *in vivo* study showed that curcumin decreased amyloid levels, plaque formation, and blocked the aggregation of fibrils in the tg2576 AD model (87). Furthermore, Tetrahydrocurcumin, a metabolite of curcumin, has a protective effect against amyloid-induced toxicity, oxidative stress, and mitochondrial dysfunction in primary hippocampal culture (88). Aside from general systemic anti-inflammatory and antioxidant activity, curcumin showed an improvement in spatial memory deficits by regulating the Brain-Derived Neurotrophic Factor (BDNF)-Extracellular Signal-Regulated Kinase (ERK) (BDNF-ERK) signaling pathway (89). Curcumin is a lipophilic compound and can cross the BBB to induce neuroprotection by increasing cyclic adenosine monophosphate (cAMP) and BDNF levels (90). Furthermore, an *in vivo* study has demonstrated that chronic curcumin infusions were associated with an increase in hippocampal BDNF expression and phosphorylated ERK levels, which was associated with enhanced memory performance. In AD models of amyloid precursor protein (APP) transgenic mice, it was found that curcumin improved memory deficits by modulating product-specific receptor (RAGE)-TLR4-NF-κB (RAGE/TLR4-NF-κB) inflammatory signaling pathway (91). In addition, curcumin reduced Beta-site APP Cleaving Enzyme 1 (BACE1) expression, a key protease in Aβ generation in dementia, through the estrogen receptor β (ERβ) and NF-κB signaling pathway in SH-SY5Y cells (92). Additionally, curcumin also demonstrated neuroprotective capacity in Aβ-Exposed THP-1 Cells by inhibiting the Mitogen-Activated Protein Kinases (MAPKs) and NF-κB signaling pathways (93). It was also seen in brain microglia cells, curcumin markedly suppressed Janus kinase (JAK)-signal transducer and activator of transcription (STAT) inflammatory signaling through activation of Src homology 2 domain-containing tyrosine phosphatase 2 (94). Due to its poor bioavailability and water solubility, many formulations have been constructed to enhance the clinical effects of curcumin (95). One formulation of curcumin and demethoxycurcumin, bisdemethoxycurcumin, improved the innate immunity and transcription of Mannoside Acetylglucosaminyltransferase 3 (MGAT3), a gene impaired in phagocytosis of Aβ plaques and Toll-like receptors in AD patients (96). In a randomized, double-blind, and placebo-controlled clinical trial, it was shown that Biocurmuax significantly improved cognitive function in older adults (97).

It has been shown that curcumin can also modulate miRs expression and activity, which mitigates the progression of neurodegenerative diseases (98). Curcumin inhibited miR-146a expression, an interleukin-1 receptor-associated kinase (IRAK)-1, IRAK-2, and NF-kB signaling pathways regulator in astroglia cells (99). Furthermore, it was found that curcumin decreased inflammatory expression of IL-1β, nitric oxide synthases (NOS), and complement factor H in the temporal lobe in AD by regulating miR-146a levels (100, 101). Studies have shown that miR-miR-26a-5p, miR-203a-3p, and miR-155-5p play a pivotal role in neuroinflammation and apoptosis through modulation of triggering receptor expressed on myeloid cells 1 (TREM-1)-DAP12-NLR family pyrin domain containing 3 (NLP3)-Caspase1-IL1B (TREM- DAP12/NLRP3/CASP1/IL1B-TLR4/NF-κB) signaling pathways, and Tau hyperphosphorylation were downregulated after curcumin administration in microglia cells (102). In addition, a nanoparticle system that co-delivers curcumin and miR-124 decreased the β-secretase, leading to inhibition of Aβ production and associated neurotoxicity (103).

#### 4.1.2 Resveratrol

Resveratrol (RSV) is a polyphenol produced by plants in response to external stressors (104). It is available in plants, fruits, grape skin, red wine, peanuts, and berries (81). Despite its therapeutic properties, it has poor bioavailability due to rapid hepatic metabolism and poor water solubility (105). To overcome these limitations, RSV can be incorporated into nanoparticles, liposomes, and polymeric particles to improve the available concentration (106). RSV has been shown to exhibit a wide range of clinically relevant properties, including antioxidant, anti-inflammatory, and anti-proliferative effects (107–109). These characteristics suggest that RSV is a potential candidate to decrease the onset of neurodegeneration and improve cognitive dysfunction.

Multiple studies show the effects of RSV on improving the progression of dementia through the modulation of different signaling pathways (110, 111). An *in vivo* study showed that resveratrol protected rats from Aβ-induced neurotoxicity by downregulating inducible nitric oxide synthase (iNOS), heme oxygenase-1 (HO-1) expression, as well as decreasing the levels of lipid peroxidation, leading to improvements in spatial memory (112). RSV also demonstrated a neuroprotective effect against β-amyloid-induced oxidative damage and memory-associated proteins in H19-7 hippocampal neuronal cells via modulation of antioxidant enzyme levels, such as superoxide dismutase, catalase, and glutathione reductase (113). Furthermore, *in vivo* and *in vitro* studies have also shown that RSV decreased the levels of glial fibrillary acidic protein, molecules related to inflammation and apoptosis, including TNF-α, IL-1β, NLRP3, cleaved caspase-3, and B-cell lymphoma 2 (Bcl-2), apoptosis regulator (Bax), as well as increased the levels of nuclear factor of kappa light polypeptide gene enhancer in B-cells inhibitor, alpha (IkBα) and Bcl-2, attenuating the cognitive impairment (110, 114, 115). Additionally, RSV mitigated lipopolysaccharide (LPS)- and amyloid-beta (Aβ)-mediated microglial inflammation by inhibiting the TLR4/NF-κB/STAT signaling cascade (116). In an *in vitro* model, using human neural stem cells, RSV showed a neuroprotective effect against Aβ-induced inflammation and oxidative stress via activation of adenosine monophosphate (AMP)-activated protein kinase (AMPK)-dependent pathways (117, 118). In addition, RSV suppressed β-amyloid-induced neurotoxicity and neuronal apoptosis via modulation of Sirtuin-1 (SIRT1)-Rho-associated coiled-coil containing protein kinase 1(ROCK1) signaling pathway in PC12 cells (119). Furthermore, RSV showed a neuroprotective effect in SH-SY5Y and HEK293 cells overexpressing APP, increasing SIRT1 activity and decreasing levels of CD147- a protein that promotes Aβ formation (109). In a randomized, double-blind, and placebo-controlled clinical trial, it was shown that RSV significantly improved cognitive function by significantly decreasing the cerebrospinal fluid Aβ40 biomarker, indicating that there are possible effects on anti-Aβ aggregation (120). Specifically, clinical trials of RSV have shown efficacy in improving cognitive impairments through modulation of pro-inflammatory cytokines and molecules related to neurodegeneration, including IL-10, IL-4, IL-12P40, IL-12P70, Fibroblast Growth Factor-2 (FGF2), regulated on activation, normal T-cell expressed and secreted (RANTES), and metalloproteinase (MMP)- 9 (110, 111). Alongside altering the levels of pro-inflammatory cytokines, RSV treatment also increased levels of serum SIRT1, improving the cognitive impairments in AD patients (121).

It has been shown that RSV can also modulate miRs expression (122). In an *in vitro* model, using human neural cells in primary culture, RSV showed a neuroprotective effect by upregulating miR-146a, which has an anti-inflammatory response in the pathogenesis of AD by decreasing levels of IL-1β and Aβ42 (123). RSV also improved learning and memory in normally aged mice, suppressing the miR-134- and miR124-cAMP response element-binding protein (CREB)-BDNF pathway (124). Additionally, RSV increased the expression of miR-2, which enhanced brain insulin signaling and resulted in decreased memory impairment (124). Similarly, RSV attenuated diabetic adipose tissue-derived extracellular vesicles-induced hippocampal Ferroptosis and cognitive dysfunction via inhibiting miR-9-3p/solute carrier family 7-member 11 (SLC7A11) axis (125).

#### 4.1.3 Sulforaphane

Another nutritional additive is Sulforaphane (SFN), which is obtained from cruciferous vegetables, such as broccoli, cauliflower, and brussels sprouts (126). It is converted from the precursor glucoraphanin into the isothiocyanate SFN by the enzyme myrosinase (127). The bioavailability is dependent on the form of supplementation and whether it was co-delivered with the myrionase enzyme (128). SFN is permeable to the blood-brain barrier, decreasing neuroinflammation and neurotoxicity (129). SFN can modulate inflammation, oxidative stress, and apoptosis to improve the outcomes of dementia (130).

SFN has exhibited a unique potential role in managing dementia development by modulating different signaling pathways (131–134). In an LPS-activated microglia model *in vitro*, SFN has been shown to reduce the levels of inflammatory mediators [iNOS, cyclooxygenase-2 (COX-2), NO, and prostaglandin E2 (PGE2)] and proinflammatory cytokines (TNF-α, IL-6, and IL-1β), through c-Jun N-terminal kinase (JNK)/activator protein-1 (AP-1)/NF-κB inhibition and nuclear factor erythroid 2-related factor 2 (Nrf2)/HO-1 activation (131). SFN has also demonstrated beneficial effects in modulating anti-inflammatory cytokines, IL-4 and IL-10, in a methylglyoxal-induced SH-SY5Y cytotoxicity model (132). In addition, SFN protected immature hippocampal neurons against death caused by exposure to hemin or oxygen and glucose deprivation through modulation of Nrf2-dependent cytoprotective genes, including NAD(P)H quinone oxidoreductase 1 (NQO1), HO-1, and glutamate-cysteine ligase modifier subunit (GCLM), which is involved in glutathione biosynthesis (133). Furthermore, a study using primary mouse brain microvascular endothelial cells has also shown that SFN protects the brain vascular system and effectively reduces ischemic cognitive deficits by increasing the adenosine triphosphate (ATP) production, expression, and activity of glucose transporter-1, and glycolysis, as well as anti-oxidative stress responses and redox signaling (135). Similarly, SFN showed a neuroprotective effect in a vascular-induced cognitively impaired model in rats through the upregulation of the Nrf2 signaling pathway, improving neuronal damage and cognition (134). Moreover, SFN was able to increase the expression of glutamate-cysteine ligase, a key enzyme in neutralizing ROS (136). Additionally, SFN improved brain metabolic stress, neuroinflammation, and cognitive impairment in rats, increasing the levels of glutathione (137). Furthermore, an *in vivo* study using AD PS1V97L transgenic mice model has demonstrated that SFN inhibited the production of amyloid-β oligomer and improved spatial learning and memory (138). In addition, a computational study showed that SFN is a potential candidate with potent and selective BACE1 inhibitory properties that play an important role in AD prevention (139).

Studies have shown that SFN modulates neuroinflammation by regulating a variety of miRs (140–143). In an *in vitro* model, using microglia, SFN showed a neuroprotective effect by inhibiting the NLRP3 inflammasome by suppressing the inflammatory miR-155 and increasing the anti-inflammatory miR-223, leading to Nrf2 signaling pathway modulation (140). It has been shown that SFN also modulates miR-17-5p, miR-16-5p, and miR-26b-5p, key miRs in neuroinflammation and the apoptosis process (141). It has been shown that in microglia of AD patients, miR-17-5p is upregulated, suppressing amyloid-β degradation (142). Similarly, miR-16-5p is upregulated in the brain tissue of 5xFAD mice, inducing neuronal cell apoptosis through direct targeting and suppression of Bcl-2 (143). In addition, miR-26b-5p expresses signals that increase Aβ levels and tau hyperphosphorylation, contributing to neurodegeneration (144).

#### 4.1.4 Ginkgo biloba

Ginkgo biloba, also known as the maidenhair tree, has leaves, seeds, and an exocarp that played an important role in traditional Chinese herbal medicine. Ingredients in the leaves include flavonoids, terpenoids, and phenolic acids (145). EGb761 is a derivative of the phytochemical Ginkgo Biloba extract (GbE) (146). It is often consumed in various forms, including tablets, guttate pills, and in teas (147). The bioavailability of the extract depends on the form that is consumed, with tablets having the highest concentrations (148). The extract can have neuroprotective effects in neurodegenerative diseases, such as dementia (149, 150). EGb 761 extract can improve hippocampal neurogenesis, decrease Aβ aggregation, and enhance neuronal plasticity (151). Overall, ginkgo biloba is anti-inflammatory, anti-oxidative, and has a stimulatory effect on neurotransmitters (152).

Ginkgo Biloba has therapeutic effects in decreasing dementia by modulating various signaling pathways involved in oxidative stress and inflammation, leading to decreased tau phosphorylation associated with Aβ deposits (149, 153). An *in vivo* study using AD Tg2576 mice has demonstrated that long-term EGb761 supplementation decreased APP levels, a key protein in the AD pathogenesis (154). Furthermore, EGb761 supplementation improved dopaminergic transmission in the prefrontal cortex, improving cognitive function (155). It has been shown that mice supplemented with Ginkgo Biloba increased the superoxide dismutase and catalase levels in the hippocampus, striatum, and substantia nigra, improving cognitive decline in rats (156). Additionally, EGb761 improved cognitive function and regulated inflammatory responses in the APP/presenilin-1(PS1) mouse through downregulating pro-inflammatory cytokines and iNOS and upregulating anti-inflammatory cytokines and Arginase-1 (Arg-1) (157). In a randomized, double-blind, and placebo-controlled clinical trial, it was shown that Ginkgo biloba significantly improved cognitive function by modulating the cholinergic synaptic transmission by impacting the activity of acetylcholine receptors (158). However, studies have shown no significant reduction in the incidence or progression of dementia among older adults receiving EGb761 supplementation (159). Similarly, a meta-analysis reported mixed results, with benefits varying by dose, duration, and study design (160). These discrepancies highlight the need for standardized protocols and long-term randomized trials to clarify the true efficacy of Ginkgo biloba in the prevention of dementia.

There is limited research on the impact Ginkgo Biloba has on miR levels in the treatment of dementia. However, the treatment with EGb761 improved cognitive dysfunction and was associated with a decrease in levels of miR-1-3p/miR-206-3p in scopolamine-induced memory deficits (161).

### 4.2 Vitamins

Vitamins are essential macronutrients that are sorted into the fat-soluble (A, D, E, K) and the water-soluble (B-complex and C), which are all pertinent for the various roles they play in human physiology. It contributes to the understanding of how individuals can utilize dietary and vitamin-based supplements to help with recovery and overall wellbeing (162). This section aims to analyze how the following vitamins- Vitamin B12, Vitamin D, Vitamin C, and Vitamin E can modulate different signaling pathways and miRs to improve memory and cognitive functions. Table 2 summarizes these mechanisms.

**Table 2:** Effect of vitamins on the pathophysiological complications associated with cognitive decline/dementia.

**Vitamins**	**Sources**	**Experimental models**	**Physiological effects**	**References**
Vitamin B12	Animal-based foods such as poultry, red meat, and fish.	*In *vitro**: Glial cells SH-SY5Y cells *In *vivo**: Rats Clinical trials: Cross-sectional study Multicenter study Case-control study Longitudinal study	↓ inflammation ↓ oxidative stress ↓ synapse ↑ PTBP1 ↓ protein levels of Stat3 ↓ pErk, and p38 ↑ pJNK ↓ apoptosis ↑ Myelination ↓DNA hypermethylated NUDT15 and TXNRD1 ↑miR-34a-5p, miR-128-3p, miR-181a-5p, and miR-204-5p ↓ miR-124a	(167, 171–174, 176, 178, 309)
Vitamins C	Citrus fruits and cruciferous vegetables.	*In *Vitro**: SH-SY5Y cells NPC cells Cortical cells *In *Vivo**: APP/PSEN1mouse. CA1 mouse model Clinical Trial: Double-blind, placebo-controlled trial	↓ Aβ aggregation ↓ptau ↓ oxidative stress ↓ inflammation ↓NMDA and glutamate levels. ↑synaptophysin levels ↑dopamine, DOPAC, 3-MT, and HVA levels. ↑ reaction time and attention scores. ↑ miR-155 levels	(180, 185, 187–194, 197)
**Vitamin E**	Nuts, seeds, vegetable oils, and green leafy vegetables.	*In *vitro**: Cortical neuron cells HT22 cells. Cerebellar granule neurons PC12 cells Microglia model *In *vivo**: Apoe-/- mice Rats	↓ Aβ aggregation ↓ptau ↓ oxidative stress ↓ inflammation ↑ MAPK-PI3K ↓COX-2 ↑ SIRT1, Nrf2, and Calstabin2 levels. ↑ spatial learning deficits and dendritic alterations. ↑ glutathione levels ↑ BDNF, CaMKII, CREB, synapsin I, SOD, and Sir2 levels. ↓ miR-122	(203, 204, 206–210, 310)
**Vitamin D**	Salmon, mackerel, milk, cereals, and sunlight exposure.	*In *vitro**: Cortical neuron cells Macrophages *In *vivo**: Rats Animal model of sclerosis Clinical trial: Double-blind, randomized placebo study	↓ Aβ aggregation ↓ptau ↓ inflammation ↓ LVSCC-A1C and LVSCC-A1D ↑ nerve growth factors ↑neural stem cell proliferation and oligodendrocyte differentiation. ↑ NT-3, BDNF, GDNF and CNTF ↑ cognitive impairment ↑ miR-9-3p	(217, 221–224, 311)

PTBP1, Polypyrimidine tract-binding protein 1; STAT3, signal transducer and activator of transcription 3; p, phosphorylation; Erk, extracellular signal-regulated kinases; JNK, Jun N-terminal kinase; NUDT15, nudix hydrolase 15; TXNRD1, thioredoxin reductase 1; NMDA, N-methyl-D-aspartate; Aβ aggregation, Beta-amyloid; DOPAC, 3,4-dihydroxyohenyl-acetic acid; 3-MT, 3-methoxytyramine; HVA, homovanillic acid; NO, nitric oxide; SIRT1, sirtuin 1; Nrf2, nuclear factor erythroid 2-related factor 2; BDNF, brain-derived neurotrophic factor; CaMKII, calcium/calmodulin-dependent protein kinase type II; CREB, cyclic AMP response element binding protein; SOD, superoxide dismutase; Sir2, silent information regulator 2; LVSCC, L-type voltage-sensitive calcium channels; NT-3, neurotrophic factors including neurotrophin-3; GDNF, glial cell line-derived neurotrophic factor; CNTF, ciliary neurotrophic factor.

#### 4.2.1 B12—Cobalamin

Vitamin B12, or cobalamin, is classified as a water-soluble vitamin (163). It is primarily sourced from animal-based foods such as poultry, red meats, fish, and dairy products (164). Vitamin B12 absorption is a complex process that has various factors influencing its bioavailability and storage (165). The active circulating forms of vitamin B12 in the body include methylcobalamin and adenosylcobalamin, while cyanocobalamin is a common supplement form (165, 166). Vitamin B12 plays a critical role in the development and aging of the brain through DNA synthesis, fatty acid metabolism, and the maintenance of the nervous system (167, 168). Suboptimal baseline serum vitamin B12 levels and a vitamin B12 deficiency have both been associated with a faster rate of cognitive decline and increased risk of dementia in AD patients (169).

It has been shown that B12 hypovitaminosis is linked to cognitive dysfunction and Alzheimer's disease gray matter atrophy (170). It has been shown that Vitamin B12 increases glial migration and synapse formation through modulation of leukocyte-common antigen-related receptor-type tyrosine-protein phosphatase (PTP-3/LAR PRTP) expression, improving neurological damage, maintaining the sensory neuron function (167). Additionally, vitamin B12 also demonstrated neuroprotective effects in hydrogen peroxide-induced apoptosis in SH-SY5Y cells by up-regulation of poly pyrimidine tract binding protein 1 (PTBP1) (171). Furthermore, an *in vivo* study has demonstrated that vitamin B12 deficiency leads to persistent brain defects by reducing transcription factor Stat3 signaling pathway, a key pathway for neuron survival (172). In addition, vitamin B12 shortages also affected upstream kinases of the Stat3 signaling pathway (phospho-Erk1/2, phospho-Src, phospho-JNK, and phospho-p38 as well as downstream target gene products (Bcl-2 and Bcl-xL), thus promoting apoptosis (172). Furthermore, in a scopolamine-induced AD model, it was found that vitamin B12 decreased hippocampal inflammation [Postsynaptic density protein 95 (PSD-95) and neurexin 1 and neuroligin] and apoptosis markers (COX-2 and activated caspase-3) and preserved pre- and post-synaptic proteins and possibly synaptic integrity in the hippocampus (173). Clinical trials for vitamin B12 are still in progress; however, it has been shown that low vitamin B12 levels are linked to cognitive impairment and dementia (174). Similarly, in another study, low vitamin B12 levels were associated with cognitive decline and AD development (175). Furthermore, lower vitamin B12 levels were associated with cognitive impairment by hypermethylation of redox-related genes NUDT15 and TXNRD1 (176). However, it has been shown that vitamin B supplementation has no significant improvements in memory or executive function among older adults (177).

Notable miRs are correlated with vitamin B12 and their impact on neurodegeneration, cognitive decline, and AD development (178). Studies have shown that B12 and other B vitamins increased the expression of different miRs, including miR-34a-5p, miR-128-3p, miR-181a-5p, and miR-204-5p, miRs relating to NFKB1, ATF3, and NR3CI expression, key genes associated with cognitive function (178). Additionally, in response to vitamin B12 deprivation, miR-124 increased, leading to the suppression of Stat3 signaling, which is associated with hippocampal neuron differentiation (172).

#### 4.2.2 Vitamins C

Vitamin C, or ascorbic acid, is water-soluble and crucial for human physiology (179). Vitamin C is obtained rapidly through diet through foods such as citrus fruits and cruciferous vegetables (180). Its homeostasis is regulated by intestinal absorption, transport to tissues, renal uptake, and urine excretion involving the Sodium-Dependent Vitamin C Transporters (181). Remarkably, vitamin C accumulates in the brain at millimolar intracellular concentrations and higher levels in cerebrospinal fluid compared to plasma (182). These elevated brain concentrations underscore their significance in antioxidant defense, neuromodulation across glutamatergic, dopaminergic, cholinergic, and gamma-aminobutyric acid (GABA)ergic systems, and protection against oxidative neuronal injury (183). Importantly, vitamin C deficiency correlates with increased risk of cognitive decline and dementia, while normalizing levels has shown potential cognitive and neuroprotective benefits (184).

Multiple studies have shown that vitamin C has exhibited an array of activity against cognitive decline and dementia progression (185–188). It has been shown that vitamin C protects SH-SY5Y cells from apoptosis and death induced by Aβ, through modulation of oxidative stress (185). Similarly, vitamin C showed a neuroprotective effect in human neural progenitor cells through the downregulation of WNT/β-catenin/ROS signaling pathway, improving cellular damage (189). In addition, vitamin C significantly protected neurons from N-methyl-D-aspartic acid (NMDA)- or glutamate-induced injury and significantly reduced cell death, proposing its role in preventing excitotoxicity in cerebral cortical neuron cells (190). Furthermore, vitamin C restores behavioral deficits and decreased Aβ oligomerization, brain oxidative stress, ratio of soluble AB42 to AB40, increased synaptophysin levels, and phosphorylation of tau at SER3 in a mouse model of AD (191). An *in vivo* study has demonstrated that vitamin B12 deficiency in the brain impairs cognition, increases amyloid accumulation and deposition, as well as oxidative stress in the APP/PS1 mouse model (186). Similarly, in another study, low vitamin C levels were associated with an increase of oxidative stress, neurodegeneration, cognitive decline, and AD development in APP/PS1 mouse model (187). Additionally, vitamin C deficiency decreased dopamine and its metabolites 3,4-dihydroxyphenyl-acetic acid (DOPAC), 3-methoxytyramine (3-MT), and homovanillic acid (HVA) release in APP/PSEN1 mice, leading to cognitive impairment (188). Moreover, vitamin C supplementation was able to increase glutamate levels, reduce neuronal apoptosis in the hippocampal CA1, pro-apoptotic Bax, and increase the levels of Bcl-2 (192). Clinical evidence further supports the potential role of vitamin C in cognitive function. In a double-blind, placebo-controlled trial, healthy young adults supplemented with vitamin C showed improved reaction time and attention scores, which were positively correlated with plasma vitamin C levels (193). In elderly individuals, long-term vitamin C supplementation improved mood, reduced memory-related errors, and enhanced global cognition, particularly in those with lower baseline cognitive performance (194). Furthermore, evidence regarding vitamin C and cognition is also mixed. Although some studies demonstrate reduced oxidative stress and improved memory with higher plasma vitamin C levels, interventional trials show minimal or no improvement in cognitive scores (195, 196).

There is limited research on the role of vitamin C on miR levels in the treatment of cognitive decline and dementia. However, it has been shown that vitamin C can regenerate antioxidants and reduce the production of inflammatory signaling molecules such as IL-6 and TNF-α, through modulation the expression of miR-155 (197). By reducing oxidative damage, antioxidants help preserve brain cells, reduce inflammation, and protect against cellular changes that could lead to dementias, memory deficits, and cognitive decline (198). More studies need to be done on this therapeutic target and the miRs involved, as there are molecular pathways with vitamin C and the progression of neurological disorders that have not been fully uncovered yet (199).

#### 4.2.3 Vitamin E

Vitamin E, a lipid-soluble antioxidant, exists in eight isoforms, among which α-tocopherol is the most biologically active in humans (200). Dietary sources such as nuts, seeds, vegetable oils, and green leafy vegetables provide vitamin E in highly bioavailable forms, often showing greater absorption efficiency than synthetic supplements (201). Upon ingestion, vitamin E is released in the small intestine, where it is emulsified by bile salts and incorporated into mixed micelles, facilitating its uptake via membrane transporters such as SR-BI and CD36 (202). Once absorbed, α-tocopherol is transported in plasma predominantly bound to lipoproteins, including very low-density lipoprotein and high-density lipoprotein, which mediate its systemic distribution and uptake by peripheral tissues (202).

Vitamin E has exhibited a crucial role in managing dementia development by modulating different signaling pathways. It has been described that vitamin E protected cultured cortical neurons from oxidative stress-induced cell death through the activation of MAPK-phosphatidylinositol 3-kinase (PI3K), decreasing the Bax/Bcl-2 ratio and apoptosis (203). Furthermore, an *in vitro* study using hippocampal HT22 cells and rat cerebellar granule neurons demonstrated that vitamin E protected neurons effectively against oxidative cell death by inducing the activity of the redox-sensitive transcription factor NF-kB (204). In addition, vitamin E prevents neuronal cell death by repressing COX-2 activity in HT22 cells (205). Additionally, the antioxidant and free radical scavenger effect of vitamin E inhibited Aβ-induced cell death in PC12 cells (206). In an LPS-activated microglia model *in vitro*, vitamin E has been shown to reduce the levels of inflammatory mediators, including TNF-α, IL-1α, and NO, by inhibition of p38 MAPK- NF-kB (207). Furthermore, an *in vivo* study showed that vitamin E supplementation improved oxidative stress, cognitive functions, in aged mice by upregulation of SIRT1, Nrf2, and Calstabin2, as well as reducing the oxidative stress, attenuating cognitive decline (208). Similarly, vitamin E supplementation prevented spatial learning deficits and dendritic alterations in aged apolipoprotein E-deficient mice, decreasing lipid peroxidation and increasing glutathione levels (209). In addition, vitamin E protects against oxidative damage and learning disability after mild traumatic brain injury in rats, increasing hippocampal levels of BDNF, Calcium/calmodulin-dependent protein kinase II (CaMKII), CREB, synapsin I, duperoxide dismutase, and Sir2 (210). Despite promising antioxidant and neuroprotective properties, clinical outcomes with vitamin E remain inconsistent. Randomized controlled trials and meta-analyses have shown no significant cognitive benefit or disease progression delay in Alzheimer's disease patients receiving vitamin E supplementation (211, 212). Variability in dosage, disease stage, and study design may explain these conflicting results, underscoring the need for well-controlled longitudinal studies.

It has been shown that vitamin E can also modulate miRs expression (213). It has been shown that miR-122 was positively and significantly correlated with inflammatory molecules [granulocyte-macrophage colony-stimulating factor (GM-CSF), interferon alpha-2 (INF-α2), IL-1α, IL-8, and macrophage inflammatory protein-1 beta (MIP-1β)] as well as with vitamin E (214). Additionally, logistic regression analysis showed that vitamin E serum levels were associated with a higher AD probability and partially mediated by miR-122 (215).

#### 4.2.4 Vitamin D

Vitamin D is another fat-soluble vitamin that is mainly obtained through sunlight exposure, dietary sources, and supplements (216). The major dietary sources of vitamin D are fatty fish (e.g., salmon, mackerel), fortified foods (e.g., milk, cereals), and supplements (vitamin D2 and D3) are primary sources (217). The presence of dietary fats, which enhances bioavailability, however, the breakdown of the dietary products in the upper gastrointestinal system, mainly in the duodenum, allows for this vitamin to be released (218). Evidence has linked adequate vitamin D levels to a reduced risk of cognitive decline and dementia, suggesting a potential neuroprotective effect (219).

It is well-known that vitamin D receptors have been found in neurons and glial cells, with their highest expression in the hippocampus and thalamus (220). It has been shown that Vitamin D downregulates the L-type voltage-sensitive calcium channels (LVSCC), LVSCC-A1C and LVSCC-A1D, and upregulates nerve growth factors, leading to a neuroprotection of cortical neurons (220). Furthermore, an *in vitro* study showed that vitamin D increases the Aβ phagocytosis by AD macrophages (221). Additionally, vitamin D supplementation ameliorated cognitive impairment and altered neurodegenerative (Aβ1-40, Aβ1-42, p-Tau, and Neprilysin) and inflammatory markers (IL-6, IL-1β, TNF-α, and NF-κβ) in the scopolamine-induced rat model (222). An *in vivo* study has demonstrated that vitamin D improves neural stem cell proliferation and oligodendrocyte differentiation by increasing the expression of important neurotrophic factors including neurotrophin-3 (NT-3), BDNF, glial cell line-derived neurotrophic factor (GDNF) and ciliary neurotrophic factor (CNTF) (223). Furthermore, in a randomized, double-blind, and placebo-controlled clinical trial, it was shown that vitamin D supplementation significantly improved the levels of Aβ-42, APP, and BACE1 in elderly patients with AD (224). Additionally, another study showed that low vitamin D concentration is associated with mild cognitive impairment status in the older population (225). However, studies have shown no significant reduction in the incidence or progression of dementia among older adults receiving vitamin D supplementation (226, 227). Thus, the role of vitamin D in the prevention of dementia may depend on baseline deficiency and population characteristics.

There is limited research on the role of vitamin D on miR levels in the treatment of cognitive decline and dementia. However, it has been shown that vitamin D upregulates miR-9-3p, which is related to neuroinflammation and memory deficits (228).

### 4.3 Metabolic modifiers/dietary interventions

The following section reviews the cognitive benefits of omega-3 fatty acids, ketogenic diets, the Mediterranean diet, and trehalose by consolidating findings from various studies. Each of these dietary modifications conveys neuroprotective effects either by preventing amyloid plaque accumulation, antioxidant properties, anti-inflammatory signaling, epigenetic modifications, or microRNA expression. Table 3 summarizes these mechanisms.

**Table 3:** Effect of metabolic modifiers/dietary interventions on the pathophysiological complications associated with dementia.

**Metabolic modifiers/Dietary interventions**	**Sources**	**Experimental models**	**Physiological effects**	**References**
Omega-3 Fatty Acids	Seeds, vegetable oils, green leafy vegetables, fish oils, fish meat, beef, lamb, marine mammal blubber	*In *vitro**: Microglia cells, BV2 cells THP-1 cells Peripheral mononuclear cells. Microvascular endothelial cells HEK293 cells SH-SY5Y cells *In *vivo**: GPR KO mouse. C57BL6mouse. 3xTg-AD mouse. 5xFAD mouse	↓ Aβ aggregation ↓ptau ↓ oxidative stress ↓ inflammation ↓BACE1 ↑ ADAM10 ↑ Akt-mTOR-S6K ↑ miR-155, miR-146, miR-21 and miR-29a-3p ↓ miR-107 ↓TRAF6, IRAK1 ↓ PDcD4 ↓ NLRP3	(236–245, 312–317)
Ketogenic Diet	Low-carbohydrate and high-fat foods	*In *vitro**: HEK293 cells. SH-SY5Y cells Monocyte cells *In *vivo**: C3HeB/FeJ mouse. C57BL6/N mouse 5xFAD mouse. APP/Ps1 mouse C57BL/6J mouse Clinical trial: Comparative studies	↓ oxidative stress ↓ inflammation ↓glutamate ↑ UCP2, UCP4, UCP5 ↑FOXO3A, MT2 ↑ PKA, ERK 1/2, Ras/Raf, PI3K, JNK ↓ NLRP3, ↓ BACE1 ↓ miR-29a ↑ miR-16-5p	(253, 255, 256, 258, 261–263, 317–321)
Mediterranean Diet	Unrefined plant foods, olive oil, eggs, fish, poultry, red meat, and red wine	*In *vitro**: Neuronal cells, SH-SY5Y cells. *In *vivo**: APPswe/PS1DE9 transgenic mouse C57BL/6J mouse APP/PS1 mouse *In *silico**: Network analyst Clinical trial: Comparative studies	↑ cognitive ability ↓ amyloid plaque accumulation ↑ ERK 1/2, PPARγ ↑ ERK pathway signaling ↑ miR-17-5p, miR-335-5p, miR-93-5p ↓ JNK3	(246, 270–274, 276, 322)
Trehalose	Plants, bacteria, and fungi	*In *vitro**: HEK293T cells H4 cells N2a cells SH-SY5Y cells *In *vivo**: Tg2576 mouse	↓ Aβ aggregation ↓ptau ↓ oxidative stress ↓ inflammation ↑ progranulin and lysosome homeostasis, ↑ spatial learning ↑memory ↑ Synaptophysin and doublecortin ↑ SIRT1, ↓ NLRP1 ↑miR-132, miR-181c, miR-539-5p	

Aβ aggregation, Beta-amyloid**;** (BACE1), ADAM10, disintegrin and metalloproteinase 10; mTORC1, mammalian target of rapamycin complex 1; TRAF6, TNF receptor-associated factor 6; IRAK1, Interleukin-1 receptor-associated kinase 1; PDcD4, Programmed cell death 4; NLRP3, nod-like receptor pyrin-containing 3; UCP, uncoupling protein; FOXO3A, forkhead box O3a; MT2, metallothionein 2; PKA, protein kinase A; ERK1/2, extracellular signal-related kinases 1/2; PI3K, phosphoinositide triphosphate kinase; JNK, jun amino-terminal kinase; SIRT1, Sirtuin 1.

#### 4.3.1 Omega-3 fatty acids

Omega-3 fatty acids, or n-3 fatty acids (n-3 FAs), are essential fatty acids with a double bond between the third and fourth carbon atoms from the methyl group, or omega (229). The polyunsaturated n-3 FAs (n-3 PUFAs) of particular interest for their health benefits in humans are α-linoleic acid (ALA), eicosapentaenoic acid (EPA), and docosahexaenoic acid (DHA), with ALA acting as a precursor molecule for EPA and DHA (230). As n-3 PUFAs are essential, they must be consumed from various dietary sources such as seeds, vegetable oils, green leafy vegetables, fish oils, fish meat, beef, and lamb (230, 231). Sources such as chia seeds or flaxseed oil are rich in ALA, which requires further modification in the body after absorption before use; however, fish oil, fish meat, and marine mammal blubber are rich in EPA and DHA, which require no modification (231). Given the limited rate of conversion from ALA to DHA and EPA, recommendations to increase dietary intake of fish and fish oil, as well as supplementation with formulations containing EPA, DHA, and triglycerides, have become increasingly popular (232, 233). Several studies have shown that those with an increased consumption of omega-3 fatty acids are at a decreased risk for developing cognitive decline or dementia (232). Increases in consumption of DHA and EPA have been shown to lower the risk of cognitive decline by around 8%−9.9% per 0.1 g/d (234). This is likely due to the anti-inflammatory and neuroprotective properties that combat the chronic neuroinflammation and amyloid plaque buildup associated with dementia and Alzheimer's disease progression, namely Alzheimer's disease (235).

Multiple studies have revealed that Omega-3 fatty acids show promising effects in mitigating dementia progression through a wide variety of signaling pathways (236–238). EPA and DHA have shown promise in suppressing the inflammatory responses of LPS-stimulated mouse microglia through the SIRT1 pathway-mediated inhibition of NF-κB signaling, decreasing the levels of pro-inflammatory cytokines IL-6, TNF,α as well as the key enzyme in the cellular metabolism, nicotinamide phosphoribosyl transferase (NAMPT) (236). In addition, DHA has also been shown to bind to the G protein-coupled receptor (GPR)120, suppressing microglia reactivity and neuroinflammation (237, 238). Furthermore, neuroprotectin D1, a stereoselective mediator derived from DHA has a protective effect against Aβ42 production by modulating key proteins involved in the processing of β-amyloid precursor protein, including peroxisome proliferator-activated receptor gamma (PPARγ)-dependent expression of BACE1, disintegrin and metalloproteinase 10 (ADAM10), and soluble amyloid precursor protein alpha (sAPPα), in 3xTg-AD mouse models and human neuronal-glial cells in primary culture (239). EPA and DHA also exhibit antiapoptotic effects in human neural cells and primary hippocampal neurons, by increasing the expression of Bcl-2, Bcl-xl, and Bcl-2-related gene expressed in fetal liver (Bfl-1) and decreasing the expression of Bcl-2-associated x Bax proteins through neuroprotectin D1 (NPD1)-mediated suppression of Aβ42 and suppression of caspase-3 and caspase-9 (240, 241). In addition, EPA and DHA suppressed the formation of tangles in AD through the attenuation of phosphorylated glycogen synthase kinase 3 (p-GSK3β) and p-Tau expression (241). Furthermore, it has also been shown that DHA leads to the activation of the Akt-mTOR-S6K signaling pathway, promoting upregulation of Tau and collapsin response mediator protein 2 (CMPR2), two important axon-related proteins in cortical neurons (242). In addition, when phosphorylated by GSK3 in primary hippocampal neurons and SH-SY5Y neuroblastoma cells, the ability of CMPR2 to assist with axonal growth is decreased. The ability of DHA to reduce the expression of GSK3 and increase the expression of CMPR2 may help to explain some of the cognitive benefits of DHA (243).

While the data is limited, studies have shown that the neuroprotective effects of n-3 PUFAs may act through modifications of miR expression (244–246). Supplementation with n-3 FAs has been shown to alleviate neuroinflammation in mouse models by upregulating miR-107 targeting piezo-type mechanosensitive ion channel component 1 (PIEZO1)/NFκB p65 signaling pathway (244). Elevation of miR-29a-3p expression as a result of treatment with EPA in murine BV-2 cells lowered the activity of nod-like receptor pyrin-containing 3 (NLRP3) inflammasomes by activating autophagy through direct interaction with MAPK8 (245). Analysis of gene expression affected by foods rich in n-3 PUFAs also shows changes in the expression of miR-17-5p, which evidence suggests could influence the regulation of APP in *in vitro* models (246, 247).

#### 4.3.2 Ketogenic diet

The classic ketogenic diet is a low-carbohydrate, high-fat diet with varying levels of protein designed to induce ketosis in the body for different health benefits (248). Ketosis occurs because the main source of energy for the body has become fatty acids, which are converted to acetyl-CoA in the liver via β-oxidation followed by synthesis with acetoacetyl-CoA to form 3-hydroxy-3-methylglutaryl-coenzyme A (HMG-CoA), the cleavage of HMG-CoA to acetyl-CoA and acetoacetate (ACA), and the eventual reduction of acetoacetate to β-hydroxybutyrate (β-HB) (249). When the levels of ketone bodies in circulation are high enough, the ketone bodies ACA and β-HB are transported across the blood-brain barrier using monocarboxylate transporters, where they are converted back to acetyl-CoA and modified in the tricarboxylic acid cycle without the use of ATP (250). The brain's usage of ketone bodies as energy, when paired with a calorie deficit, provides several neuroprotective benefits, such as a reduction in the damage and dysfunction of neurons, an increase in neuroprotective factors, an improvement in mitochondrial function, and a decrease in neuroinflammation (251).

Ketogenic diets have been reported to have protective roles in dementia progression; however, the neuroprotective effects are not yet fully understood. It has been shown that one of the mechanisms related to Ketogenic diets is related to decrease in oxidative stress levels (252). In an *in vitro* study in neocortical neurons, ketone bodies significantly inhibited oxidative stress through mitochondria modulation, a key pathway that contributes to vascular dementia and AD progression (253). Similarly, in another study, Ketone bodies were able to increase the rate of reduced nicotinamide adenine dinucleotide (NADH) oxidation without altering glutathione levels, protecting against glutamate toxicity and ultimately, neuronal death (254). In addition, while ketone bodies showed a reduction in the amount of reactive ROS produced by neuronal mitochondria, specifically in complex I, these molecules also increased the expression of uncoupling proteins (UCP)**-**2, UCP-4, and UCP-5 as well, further decreasing the level of ROS, leading to neuroprotective effects in the hippocampus (255). Another study, which evaluated the effects of β-HB treatment on mice, showed that β-HB also acts as a histone deacetylase inhibitor, suppressing oxidative stress by modulating the expression of forkhead box O3a (FOXO3A) and metallothionein 2 (MT2) (256). Additionally, insulin sensitivity, which is impaired in AD development, is also affected by ketone bodies through modulation in the expression of protein kinase A (PKA), ERK1/2, Ras/Raf, PI3K, and JNK in neurons and astrocytes from C57Bl6/N mice (257, 258). The ability of ketone bodies to affect insulin sensitivity, as well as act as an alternate energy source to glucose, may help ameliorate the increased cerebral amyloid burden that correlates with the high-glycemic state seen in Alzheimer's disease (259, 260). Additionally, comparative studies of the brains of AD patients and cognitively normal aged adults showed impaired uptake of glucose, but not ketone bodies, further supporting the role of ketone bodies as an alternative energy source for the brain (261). Clinical trials have also shown improvements in daily functioning, quality of life, and memory for Alzheimer's patients consuming a ketogenic diet (262, 263).

There is limited research on the impact of Ketogenic diets on miR levels in dementia progression.

#### 4.3.4 Mediterranean diet

The Mediterranean diet (MD) refers to the typical dietary patterns of those living in parts of Greece and Italy in the 1960s; the diet is characterized by unrefined plant foods, olive oil, eggs, fish, poultry, small amounts of red meat, and moderate red wine intake (264). The diet has long been studied for its health benefits, including reducing the risk of dementia and AD development (265, 266). Some of the potential health benefits are due to the high amount of omega-3 fatty acids consumed in the diet, the benefits of which have been addressed in this review (246). The diet is also rich in polyphenols and B, C, D, and E vitamins, whose neuroprotective effects have also been reviewed (246). Other beneficial effects of the MD come from the consumption of magnesium-rich foods, which show cognitive benefits through alterations of the calcium: magnesium (Ca:Mg) ratio (267). Overall, the nutrients consumed from the classic MD confer antioxidant effects, anti-inflammatory effects, and inhibition of Aβ deposition, reducing the risk of dementia and AD development (268, 269).

The MD is rich in calcium and magnesium (267, 270). Lowering the Ca:Mg ratio by Mg supplementation improves cognitive function, partially through 5-mC modifications of two CpG sites in the APOE gene (271). In addition, magnesium itself directly inhibits NMDA receptors, a receptor that plays a critical role in Alzheimer's pathology, through glutamate excitotoxicity (272). In another study, magnesium was able to modulate the cleavage of Aβ precursor protein to the non-amyloidogenic pathway through activation of the ERK1/2 and PPARγ signaling pathways in APPswe/PS1DE9 transgenic mice (mouse model of AD), improving cognitive decline (273, 274). In addition, magnesium ameliorated cognitive deficit by attenuating hippocampal neurogenesis impairment in an APPswe/PS1dE9 mouse model of AD (275).

Comparisons of gene regulation and expression in the MD show upregulation of three miRs: miR-17-5p, miR-335-5p, and miR-93-5p (246). Analysis of gene expressions affected by foods rich in n-3 PUFAs also shows changes in the expression of miR-17-5p, which evidence suggests could affect the regulation of APP in *in vitro* models (246, 247). miR-335-5p is inversely correlated with the expression of JNK3, a protein associated with Aβ accumulation (276). The role of miR-93-5p in dementia has not yet been fully elucidated, but studies show that it is upregulated in the brain tissue of patients with AD (277–279).

#### 4.3.5 Trehalose

Trehalose is a disaccharide comprised of two glucose molecules, commonly found in plants, bacteria, and fungi (280). Trehalose is useful in cryoprotection and protein stabilization and has been found to modulate metabolism in cancer cells, has shown influence over several signaling pathways, and its neuroprotective effects are still under investigation (281). Studies have shown that trehalose is capable of acting as an autophagy activator, inhibiting the accumulation of proteins, and influencing different pathways involved in oxidative stress, protein folding, and neuroinflammation regulation (282).

It has been shown that trehalose has a protective effect on dementia and AD development (283–285). An *in vitro* study showed that trehalose upregulates progranulin, a secreted growth factor important for neuronal survival and lysosome homeostasis, improving dementia in human cells H4, N2a, and SH-SY5Y (283). Furthermore, an *in vivo* study demonstrated that trehalose improved spatial learning and memory, levels of synaptophysin (synaptic vesicle protein), and doublecortin (marker of neurogenesis) in the hippocampus and cortex in the Tg2576 Alzheimer's mouse model (284). Similarly, trehalose significantly restored memory, reduced neuroinflammation, and preserved neuronal density in the hippocampus and frontal cortex in the Aβ-induced murine model of AD (285).

Studies have shown that Trehalose modulates neuroinflammation by regulating a variety of miRs (286–288). The expression of miR-132, as well as the expression of SIRT1, is upregulated by trehalose in the hippocampus, reducing neuroinflammation (286). miR-132 also directly inhibits phosphatase and tensin homolog deleted on chromosome 10 (PTEN), forkhead transcription factor O3a (FOXO3a), and histone acetyltransferase p300 (P300), which are pro-apoptotic proteins of the Akt pathway (289). miR-181c is upregulated by trehalose and alleviates the progression of AD via targeted downregulation of NLRP1 (287, 290). Additionally, miR-539-5p is upregulated by trehalose, also contributing to neuroprotection (288). miR-539-5p inhibits Aβ accumulation, tau phosphorylation, oxidative stress, as well as apoptosis by directly targeting APP, Caveolin 1, and GSK-3β. The neuroprotective effects are conveyed via regulation of the PI3K/Akt/GSK-3β pathways as well (291).

## 5 Translational and clinical perspectives

As beneficial as the covered nutraceutical interventions may be for alleviating dementia symptoms and progression, there are many barriers to consider when implementing nutraceutical-based strategies in rural settings. Those living in rural areas face persistent structural challenges, including limited affordability, reduced proximity to healthy food sources, insufficient nutritional education, and entrenched stigmas or misconceptions surrounding health programs.

A significant contributor to rural nutritional disparity is the prevalence of food deserts, where individuals consume lower-quality diets compared to their urban counterparts (13). The Supplemental Nutrition Assistance Program (SNAP) is a program designed to assist low-income households with the acquisition of food; however, it does not adequately address rural-specific constraints (292). Research indicates that even SNAP recipients in rural Appalachia experience disproportionately high food insecurity relative to non-recipients, underscoring systemic gaps in reach and effectiveness (292). Grocery stores that accept SNAP benefits are also less likely to have diverse food options, and rural farmers' markets are less likely to accept SNAP benefits (293). Transportation barriers, administrative complexity, and reduced program accessibility (294, 295). Educational efforts, specifically those targeting schools, can play a vital role in shaping long-term health behaviors. Poor childhood dietary habits are associated with chronic conditions in adulthood (296, 297). Structured school lunch initiatives requiring fruit and vegetable intake have shown the capability to reduce childhood obesity, which in turn reduces the risk of obesity in adulthood, a major risk factor for poor health outcomes like dementia (298, 299). Beyond SNAP, community-based interventions that modify both education and food environments have proven effective (300). In addition to improving SNAP coverage for rural contexts, expanding educational outreach and increasing farmers' market participation may strengthen program impact (293, 300). These initiatives underscore the importance of local engagement and multilevel interventions in promoting healthier dietary behaviors in underserved rural regions.

This review encompasses several dietary components and modifications as interventions for dementia and its associated complications, particularly in rural populations. However, effective dietary strategies to reduce dementia risk must go beyond generalized recommendations. While lifestyle modifications are associated with lower dementia risk in older populations, isolated supplements or single modifications can only partially counteract genetic susceptibility and environmental exposures (301). The most effective treatments for dementia are multimodal and personalized to account for the patient's genetic predispositions and environmental factors (302, 303). Only 12.2% of Americans are metabolically healthy, with rural elderly populations disproportionately affected by metabolic syndrome (303, 304). In order to properly implement dietary modifications to mitigate dementia risk, symptomology, and progression in elderly rural populations, the modifications must be multifactorial, personalized, and economically accessible through public health policies and community-based initiatives.

The novelty of our review is in linking population-level determinants with molecular mechanisms to propose a unified model of dementia risk. This model positions nutraceutical strategies as part of a broader approach to address health challenges in rural populations. Connecting social factors such as food insecurity and environmental toxin exposure to microbiota-driven neuroinflammation, it supports the development of targeted interventions at different levels, including public health actions, community programs, and molecular therapies.

## 6 Conclusions

**Figure 2:**
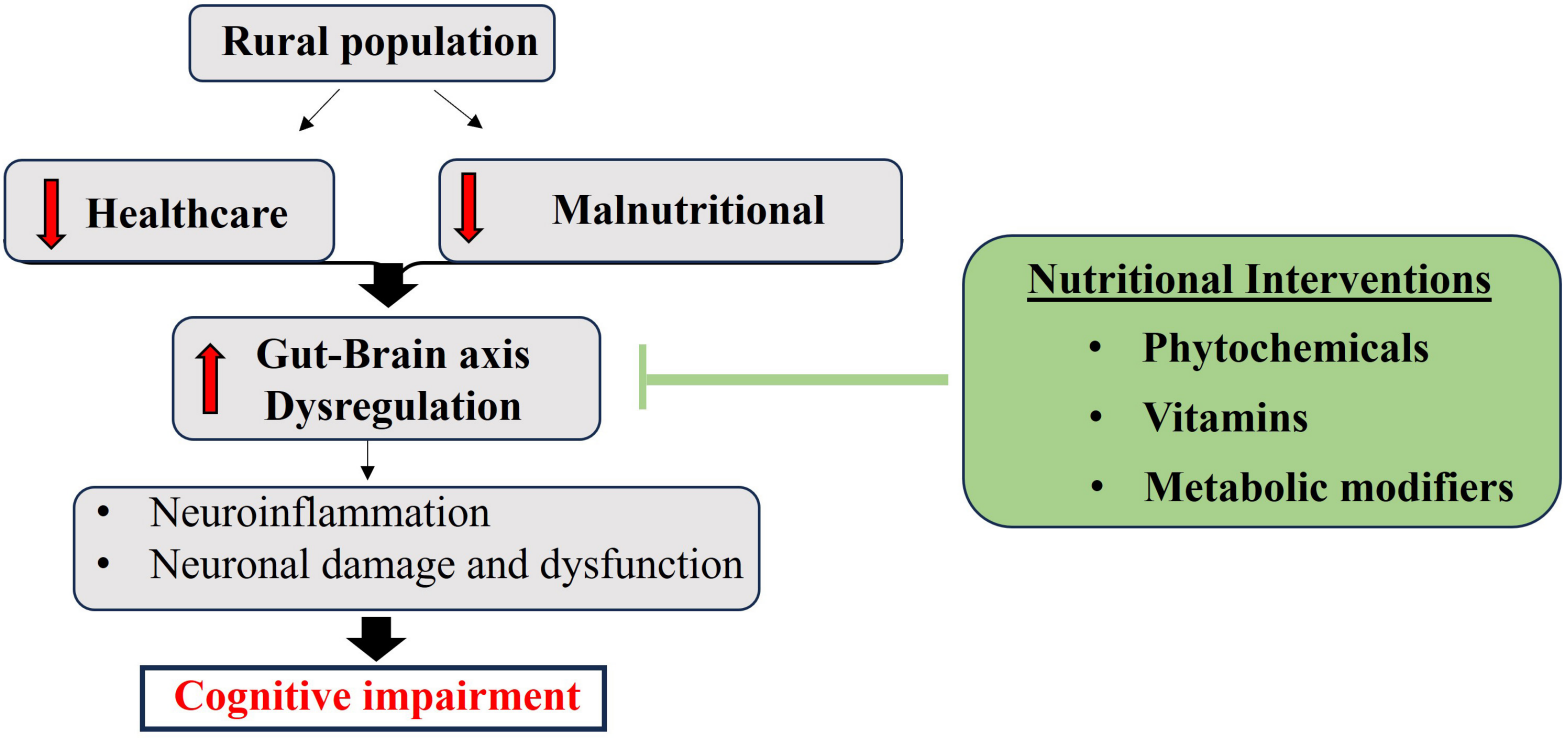
Schematic representation showing the nutritional strategies against dementia in rural populations. Image proposed by the authors.

In summary, this review describes the pathogenic progression of dementia through the gut-brain axis and the importance of nutraceuticals for its possible mitigation (Figure 2). As presented, dementia is a multifactorial disorder influenced by genetic, environmental, and lifestyle factors, many of which are disproportionately concentrated in rural areas. We emphasized that rural populations are vulnerable to cognitive impairment due to nutritional inadequacies, limited access to preventive healthcare, and adverse social determinants of health. Additionally, we showed that the gut-brain axis has emerged as a central mediator linking these factors to neuroinflammation and cognitive decline. Furthermore, the nutraceuticals presented have demonstrated efficacy in preclinical and clinical models by attenuating oxidative stress, modulating inflammatory responses, and regulating miRs involved in neuronal survival. We believe that the studies consolidated in this review illustrate the therapeutic potential of specific nutraceuticals in mitigating cognitive impairment in rural populations through modulation of the gut-brain axis. However, translating these findings into clinical and public health practice remains challenging. Economic constraints, geographical barriers, and inequitable access to healthcare services continue to limit implementation—particularly in rural settings with insufficient infrastructure. Successful integration of precision nutrition into dementia prevention and management will require coordinated interdisciplinary efforts, evidence-based clinical guidelines, and policy-level support. Importantly, the strategies discussed here align with the United Nations Sustainable Development Goals (SDGs), particularly SDG 3 (“Good Health and WellBeing”) and SDG 10 (“Reduced Inequalities”), by promoting equitable access to nutrition-based interventions and reducing disparities in cognitive health outcomes. Addressing these barriers is essential to ensure that nutraceutical-based strategies are accessible, scalable, and effective across diverse populations. Future work should focus on integrating precision nutrition into rural healthcare frameworks, strengthening the gut–brain axis as a therapeutic target, and supporting long-term dietary interventions to promote cognitive resilience in vulnerable communities.
